# “How can you think about losing your mind?”: A reflexive thematic analysis of adapting the LivDem group intervention for couples and families living with dementia

**DOI:** 10.1177/14713012241272805

**Published:** 2024-09-17

**Authors:** Natasha S. Woodstoke, Beth Winter, Emily Dodd, Richard Cheston

**Affiliations:** School of Social Sciences, 1981 University of the West of England, Bristol, UK; School of Social Sciences, 1981 University of the West of England, Bristol, UK; School of Health and Social Wellbeing, 1981 University of the West of England, Bristol, UK; School of Social Sciences, 1981 University of the West of England, Bristol, UK

**Keywords:** Alzheimer Disease, psychosocial intervention, psychological adjustment, emotional adjustment, Psychotherapy, psychology

## Abstract

**Introduction:** Despite the psychological challenges that dementia creates, comparatively little attention has been paid to how individuals or families can be helped to adjust to dementia. One of the few interventions to do this is the Living well with Dementia (LivDem) post-diagnostic course. LivDem focuses on supporting individuals to talk more openly about their dementia. However, while family supporters attend preliminary and follow up sessions, their role is limited and finding a way for them to be more actively involved might enhance the impact of the intervention and make it more flexible. We therefore set out to explore how the current LivDem intervention could be adapted for couples and families. **Method:** We completed eleven semi-structured interviews and focus groups with four groups of stakeholders: people living with dementia and their families: LivDem facilitators; researchers in this area; and psychotherapists with experience of working with couples or families living with dementia. Interviews were transcribed and analysed using reflexive thematic analysis. **Results:** Four main themes were generated: “Hear the impact on everybody”; People who are “ready to do that”; “It’s such a fine line”; and “You deal with it in your family”. Participants emphasised that the intervention needs to be delivered by willing and skilled facilitators to people who are ready to talk in their family context; and this intervention needs to be embedded within connected services. **Conclusions:** Stakeholders felt that it would be possible to adapt the LivDem model for couples and families so long as a number of conditions were met. An adapted family or couple version of LivDem has the potential to facilitate improved adaptation to dementia and to be incorporated into dementia pathways and delivered with the NHS and the voluntary sector. Further research is needed to establish the feasibility of such an intervention.

## Introduction

It’s kind of unthinkable, isn’t it? I mean, you know, everyone says death is unthinkable, but I think dementia is a bit unthinkable too. And how can you think about losing your mind? (Leah, psychotherapist)

Dementia represents an existential threat to a person’s identity, creating profound challenges to those who are affected by any form of this disease (Cheston & Christopher, 2019). These challenges occur not only in the context of changes in neurological functioning but also within relationships which are adapting to accommodate both impairments arising from the disease process itself and the wider social positioning of self (Sabat, 2008a; 2008b). Given the highly threatening nature of dementia it is understandable, therefore, that people respond to receiving a diagnosis in many different ways (Cheston, 2022). While some are shocked and react with disbelief and anger (Byszewski et al., 2007), others say that it confirms their suspicions (Derksen et al., 2006) and are relieved that their symptoms have an explanation (Pratt & Wilkinson, 2003).

Despite evidence that for many patients with chronic health conditions, being able to talk about their illness helps adjustment (Badr & Acitelli, 2005; Badr et al., 2008), many dementia services provide limited support after a diagnosis and often do not routinely provide opportunities for people to discuss their dementia diagnosis (Royal College of Psychiatrists, 2022). In addition, social pressures and changes in relationships may inhibit families or couples from initiating a discussion about the condition. Indeed, a survey of over 2,000 people from 54 different countries found that a clear majority of both respondents with dementia (over 75%) and carers (64%) believed that there were negative associations, amounting to stigma, towards dementia in their country (Batch, Mittelman & Alzheimer’s Disease International, 2012). Roughly a quarter of the 127 people who were living with dementia and completed the English language part of the survey reported that they had hidden their diagnosis of dementia from others. One respondent contrasted the response of others to her dementia with the more compassionate reactions to her cancer diagnosis: “*Being a cancer survivor, I know that I was constantly asked how I was doing while I was going through treatment. With Alzheimer’s, no one asks*”. While there have been welcome steps towards encouraging a wider awareness of dementia, nevertheless there is a continuing social stigma around this condition (Lion et al., 2020; Nguyen & Li, 2020). As a Dutch study of the diaries kept by sixteen people living with dementia has highlighted, many people living with dementia are acutely aware of how they have changed in “*the eyes of others*” and often feel scrutinized by inquisitive, negative and disapproving looks (van Wijngaarden et al., 2019). This awareness of a potentially negative response from others can impact on the persons’ willingness to discuss their illness, including with those who are closest to them.

Despite the emotional and psychological challenges that dementia creates, until recently comparatively little research or clinical attention has been paid to how services can support individuals or families to talk more openly about and adjust to dementia. Indeed, some research has suggested that enhanced adjustment in the form of greater awareness of dementia may be associated with a worse quality of life (e.g., Alexander et al., 2021). Nevertheless, there is a growing consensus both about the importance of supporting people to adjust to dementia (Brooker, Dröes, & Evans, 2017) and the psychological benefits of doing so (Brooker et al., 2014). There is also some evidence that enhancing hope, increasing social support and improving self-esteem will facilitate more adaptive coping and improved adjustment (Cotter et al., 2018; Cheston et al., 2015; Wolverson et al., 2010). Moreover, there is a growing body of clinical and research work focused on applying psychotherapeutic constructs to enhance the ability of couples and families to talk to each other about the dementia (e.g., Auclair et al., 2009; Cheston, 2022; Epstein et al., 2007; Larochette et al., 2020; Whitlach et al., 2017).

One of the few interventions to specifically focus on enhancing adjustment is the Living Well with Dementia (LivDem) post-diagnostic course (Cheston & Marshall, 2019). LivDem is an eight-week group intervention aimed at helping people to talk more openly about dementia and adjust to their condition. Sessions last for 90 minutes and are led by two facilitators who are typically nurses, occupational therapists, psychology assistants or trainee clinical psychologists, often under the supervision of clinical psychologists. LivDem is currently delivered across over 50 sites in the NHS and voluntary sector in the UK, as well as in Ireland, Italy and Japan.

The course takes a deliberately slow pace, initially focussing on the symptoms of cognitive impairment, the impact of this and ways in which participants cope with their feelings. Only when the group has formed and is ready to discuss more emotionally sensitive material, do course facilitators introduce questions around sharing their diagnosis with others and look in detail at the diagnosis, its treatment and prognosis. Finally, LivDem looks at ways of living well with dementia, including making decisions about the future, and the importance of staying as active as possible (Cheston, 2022).

LivDem is solely aimed at people living with dementia. While carers attend a session at the start and end of the intervention, LivDem facilitators have highlighted (Cheston et al., 2024) the potential benefits for involving family supporters more actively in the intervention. We therefore set out to explore whether the LivDem model could be adapted to support couples and families without compromising the underlying principles of creating a safe space to talk about dementia at a pace that is appropriate for participants (Cheston & Marshall, 2019). In this paper we report the views of participants from four stakeholder groups (people living with dementia and their families; LivDem facilitators; academics who had researched how families living with dementia adjusted to the illness; and psychotherapists with experience of working with couples or families living with dementia) about the challenges and opportunities for an adapted form of LivDem in enabling adjustment.

## Method

The study received approval from the ethics committee of the faculty of Health and Applied Sciences of the University of the West of England^1^. We obtained written consent from participants prior to interviews. All participants, including those living with dementia, had capacity to provide informed consent^2^.

### Recruitment

We recruited 26 participants from four different groups (people living with dementia and family supporters; LivDem group facilitators; dementia researchers; and psychotherapists) to consult on their views of adapting LivDem (see Table 1). None of the people living with dementia or their supporters had previously taken part in a LivDem course.

**Table 1.** Recruitment of stakeholders.

Number of participants	Recruitment method
People living with dementia (n = 3) and family supporters (2)	Dementia Action Alliance and the Join Dementia Research register
Current LivDem group facilitators: UK - NHS or voluntary, community and social enterprises sector (9), Ireland (1), Italy (1)	Via database of professionals who had attended LivDem group facilitator training
Dementia researchers (6)	Direct contact with UK academics (via email or social media) carrying out research into either the impact of dementia on couples or on delivering psychosocial interventions
Couples’ psychotherapists (4)	Purposive recruitment of psychotherapists with experience of working with couples affected by dementia

### Responding to distress

In order to minimise distress for participants who were living with dementia and their families we took a number of precautions. All potential participants living with dementia and their families met a member of the project team before the focus groups occurred to ensure that that taking part was appropriate for them as a family or as individuals. Both RC and BW are experienced clinicians who have been delivering couples therapy to people living with dementia and their families for many years and were used to discussing difficult issues in a sensitive and compassionate manner. We also created a ‘dealing with distress’ protocol which set out a step-by-step procedure for responding to distress (although this did not need to be used during data collection).

### Data collection

We held eleven focus groups and face-to-face interviews, with separate meetings for participants from the four groups. All data collection events took place online and were recorded. Using the Consolidated Framework for Implementation Research (CFIR) we created a topic guide (Appendix One) focusing on five domains of the adaptation process: the intervention, the outer and inner settings, the individual and the process (Damschroder et al., 2009). The topic guide for each of the four groups focussed on the domains that were most relevant to their knowledge and experience base. Whilst the research team was based in the south-west of the UK, workshops included participants from the rest of the UK as well as in Ireland and Italy.

### Data analysis

Interviews were transcribed orthographically using the notation system suggested by Clarke and Braun (2013) then anonymised. Reflexive thematic analysis (Braun & Clarke, 2019, 2021) was used to analyse these interviews because this approach fits with our stance that as researchers we play an active role in knowledge production, as well as allowing data collection and analysis that “respected and expressed the subjectivity of participants accounts” (Byrne, 2022).

Reflexive thematic analysis is an approach to qualitative inquiry which involves identifying, analysing, and reporting patterns within data. It has six phases for analysis, which are recursive and iterative, rather than linear: familiarising yourself with the dataset; generating initial codes; generating themes; reviewing potential themes; defining and naming themes; and producing the report. The initial coding and generation of themes, supported by NVivo, was led by BW and these were explored in collaboration with NW, with the aim of achieving richer interpretations of meaning. These were then reviewed, defined and named through discussion between RC and NW. NW led on the initial draft of the report, which was then revised by BW and circulated to all authors for further comments.

### Reflexive statement

All of the authors are psychologists: NW and RC are clinical psychologists; BW is a counselling psychologist; and ED is a research psychologist. Along with Ann Marshall, RC is the co-author of the LivDem manual for groups whilst ED has worked on developing the LivDem approach for a number of years. In contrast NW and BW were employed on short-term contracts and do not have similar emotional or practical investments in the longer-term outcomes from LivDem. As an analytical team, therefore, we attempted to be mindful of the balance between RC and ED’s commitment to the model of group work with a position of open enquiry as to whether the model could be adapted and if so, what such an adaptation might look like. NW and BW tended towards a constructionist epistemology and in their analysis held that “meaningfulness is highly influential in the development and interpretation of codes and themes” (Byrne, 2022), rather than simply looking for the recurrence of important information. ED has an interest in realist methodology and therefore brought a focus on uncovering, generating and testing theories about the underlying causal mechanisms that are activated in different contexts that may or may not produce intended and/or unintended outcomes. As a team, we were influenced by the social model of disability (Oliver, 1983), that is that we are “not disabled by our impairments but by barriers we face in society” (Oliver, 2013).

## Results

**Figure 1.**
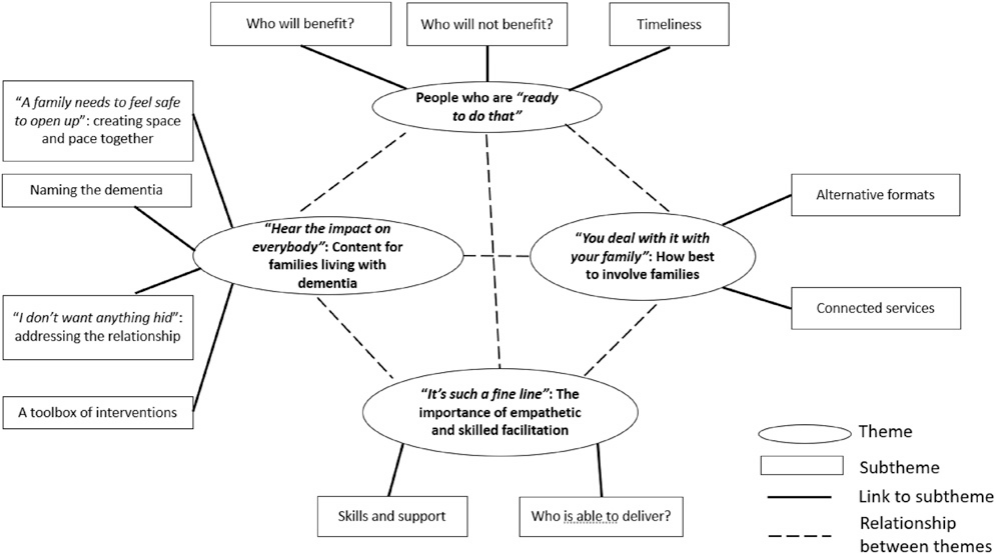
Finalised thematic map of themes and subthemes.

We generated four closely connected main themes and twelve subthemes from the data (see Figure 1). As illustrated in the thematic map (Byrne, 2022), we interpreted participants as arguing that for an intervention to be effective, certain conditions needed to be met: the intervention needed to be delivered by willing and skilled facilitators to people who were ready to talk; and this intervention, in whatever format, needed to be embedded within connected services. We will now describe these themes and subthemes further, with illustrative pseudonymised quotes from participants.

### *“Hear the impact on everybody”:* Content for families living with dementia

All three participants who were living with dementia highlighted the emotional impact of dementia on both them and their relationships:

It is a real punch, to find out these things and you don't want to believe it, you know, nobody wants to believe it. (Robert, person living with dementia)

Frustrating, very frustrating, and scary. (Grace, person living with dementia)

Because life with dementia is hard, you know? No, no getting away from that. And there's no escaping the fact that it has impacted upon our relationship. (David, person living with dementia)

Placed within this context, participants framed their comments not in the conceptual terms of whether there was a need to support couples and families to adjust after a diagnosis, but in the more practical terms of just how this support might work.

#### “A family needs to feel safe to open up”: creating space and pace together

All of the participants recognised that helping the couple or family to talk together about the impact of dementia would inevitably necessitate engaging with often difficult emotions. Drawing on their experiences with LivDem groups, two facilitators stressed the importance of allowing a safe, therapeutic space for the family to communicate openly about what was happening. Sarah (LivDem facilitator) spoke about the importance of “*giving people a space to talk about some of the challenges and the dilemmas that that they face”,* while Phoebe (LivDem facilitator) said: “*It’s about talking about new things that frighten them, using the ‘D’ word, opening things up for discussion*”. Other participants referred to the need to pace the speed at which emotionally threatening aspects of dementia were addressed. Iris, a psychotherapist, spoke of the need to “*slowly start to open things up and talk about changes they’ve noticed and how they feel about those*”. For these participants, who tended to be either LivDem facilitators or psychotherapists, helping couples or families to adjust to dementia necessitated framing their distress within a therapeutic context:

What’s it been like having this diagnosis or this news? What they feel about it, what they imagine each other feel about it?… I suppose at the end of the day, you are wanting to, for there to be a space to mourn, acknowledge the change or the loss. (Leah, psychotherapist)

At the same time, participants from all four of our stakeholder groups recognised that while the emotional and psychological challenges that couples faced might be similar, “*the journey and needs are very different for the person living with dementia and carer”* (Nick, researcher). Thus, Mia articulated a view held by a number of participants that any intervention that aimed to help couples or families to adjust together, would have to find a way to address occasions where whilst the person living with dementia and their families may be on a similar journey, at times they were on parallel, rather than overlapping, lines with differing needs. She described the positions of the person with dementia and their partner as being:

Like two rails that never meet because they (the person living with dementia) spoke freely and openly about their experience in the groups, but then they didn't speak about it at home … People with dementia really ask for our help to communicate with their care partner because … it really is a very strong existential threat, it's very frightening and the people with dementia can speak about it, but … the care partner says ‘Oh no, but it won't. Don't worry. It's going to be fine’. And that's a coping mechanism, obviously, because it's just too painful to think about that for them. (Mia, LivDem facilitator)

These concerns led some participants, especially those who were couples’ psychotherapists or LivDem facilitators, to emphasise the need to provide a safe therapeutic space where people living with dementia could be empowered to articulate their feelings without being over-shadowed by their partners and families.

#### “I don’t want anything hid”: addressing the relationship

Several participants acknowledged that while there were differences between the needs of the person with dementia and their family, nevertheless the intervention should focus on enhancing their relationship. In this sense there was more in common between the person with dementia and their family members than divided them. Although they might have different needs, participants acknowledged it was important to have a place where changes in their relationship can be usefully explored:

I guess I wouldn’t use necessarily the word ‘relationship’, I’d use the word ‘together’ and that also there’s a sort of unwelcome third person, a ‘third issue’ that’s arrived which is the dementia. (Phoebe, LivDem facilitator)

All the participants living with dementia, stressed the importance of having a place to talk about the impact of this “*unwelcome third person”*. For David, addressing the impact of dementia on his relationship with his wife meant reframing her role away from the commonly used description of her as a ‘carer’ to one in which she was his ‘supporter’:

So, a supporter doesn't take over: a supporter doesn't belittle you, doesn't make things awkward or difficult for you, doesn't speak for you … whereas a supporter, it's got much less, I almost want to use the word, responsibility. (David, person living with dementia).

In a similar way Grace was concerned that she should be allowed to talk for herself, rather than have decisions made by her partner James:

But I still want to know what's going on. What's being said about me? Because it's me that they're talking about, and I'm not a wooden box - I’m a sentient person ... I don't want anything hid. (Grace, person living with dementia).

For Robert, having a space to talk would enable others within his wider family to understand how dementia was impacting on him:

Noise starts to get to me ... but the children understand that if I do go away then I am to be left alone … explaining to grandchildren for example why I don’t want a cuddle, why I forget their names. (Robert, person living with dementia).

Later, in theme three (*“It’s such a fine line”*) we will explore in more depth an issue that was certainly implicit and occasionally explicit within stakeholders’ accounts: namely whether it would be possible to provide facilitators with the skills required to allow both the person with dementia and their supporter to have a voice, whilst at the same time supporting their relationship.

#### A toolbox of interventions

As well as facilitating discussions together about how dementia was impacting on the relationship, participants brought up other areas that they thought an intervention would need to address. This included a wide range of content that should be addressed as well as techniques to do so, much like a “*toolbox*” (Claudia, researcher) where facilitators have skills to choose from according to the needs of the couple or family they are working with. These included practical information about symptoms of dementia, financial benefits and planning for future challenges. As Robert (a person living with dementia) put it, people need “*more than a leaflet in hand*”.

While some participants framed the discussion of dementia as a simple exchange of information, others raised the emotional implications of this process of engagement with dementia – especially when this related to a future characterised by deterioration. A discussion between Grace (living with dementia) and James (her supportive partner) illustrates this dilemma in which while it is important to be prepared for the future, thinking about this involves addressing uncomfortable topics:

James: (Thinking about the future) is scary, and she (Grace) would rather just let things be. And as things come up, then we deal with them. Now the advice we've been given is that we look ahead more than that really, so that things don't take us by surprise.

Grace: I don’t want to because it's going to be horrible.

James: So that's where if people come in to talk about that sort of thing, then they're going to have to be very sensitive about whether people even want to. And how to give advice in that sort of scenario.

Grace: Yes, and at the moment I'm dreading the process of getting worse. And I think that's where I am at the moment.

As Leah, a psychotherapist remarked “it's kind of unthinkable, isn't it? I mean, you know, everyone says death is unthinkable, but I think dementia is a bit unthinkable too. And how can you think about losing your mind?”. Perhaps unsurprisingly, then, some participants described interventions that might help couples to manage their distress, such as “the types of things you can do to help calm things down” (Phoebe, LivDem facilitator).

#### Naming the dementia

A central feature of the LivDem course involves group participants discussing the advantages and disadvantages of telling other people about the diagnosis. These discussions frequently touch on wider issues around participants’ willingness to identify themselves as having dementia, social isolation and on the stigma surrounding dementia. In thinking about how to adapt the LivDem group curriculum for work with families, some stakeholders also referred to the wider social context:

I think you know, the more you use the word, the more people combat it and feel comfortable with it, the more fear is taken out of it. (David, person living with dementia)

Perhaps because of the meanings ascribed to dementia within society, some participants expressed concerns at talking about their dementia with others. Rather, the prospect of sharing the diagnosis with others raised difficult issues:

[I’m] afraid of what they’ll think of me, like … letting them [family] down. (Robert, person living with dementia)

That's not how she [her mother] would want to identify herself. That's not, it's like I don't think she would accept if we said ‘well, you know, let's go and meet other people with dementia’. (Eva, family supporter)

Some of the participants who have personal experience of dementia seem to be referring to an aspect of shame – which can be understood as part of the internalised, private consequences of the stigma that dementia can carry (Aldridge et al., 2019; Cheston, 2005; Lopez et al., 2020). This reluctance to be publicly identified as having dementia is one of the main reasons given by potential participants for not attending a LivDem course (Cheston, 2022).

### People who are “*ready to do that”*

The aim of the LivDem course is to help people living with dementia to talk more openly about their dementia. However, given both the threatening nature of dementia and the neurological damage that it creates, several participants emphasised the importance of distinguishing between people who might benefit from this intervention and those for whom it might cause distress.

#### Who might benefit?

Participants were encouraging about the potential for LivDem to meet an important need for families or couples where there otherwise wasn’t the space to address the complex and psychological challenges of the diagnosis. This was especially important where there was a particular need for the person to explore the diagnosis with their partner or other members of their family:

I mean, what stops people from taking in sort of difficult life changing kinds of information? I guess the quality of the relationship, the amount of capacity. But so much depended on what it landed on, in people's capacity to face loss. And to feel that whether or not they're going to be looked after and cared about. (Leah, psychotherapist)

An adapted version aimed at couples or families might also complement the existing group-based course. A more flexible intervention would have the potential to fit the needs of people with dementia who: don’t like or who are “*too outspoken”* for groups (Phoebe, LivDem facilitator); cannot get to groups (David, person living with dementia); or for those people whose cognitive or physical impairments prevented them from taking part in a group (Janet, researcher). A family-based intervention could be more easily tailored for younger families affected by early onset dementia or delivered online to bring together people living with dementia who were physically separated from other members of their family (David, person living with dementia):

Get away from NHS one size fits all service and actually what we need is the individual assessment and then a range of different offers that we have based on what people's needs are. (Lucy, researcher)

Thus, a more flexible intervention focused on the needs of a family unit, was seen to have potential benefits by all four stakeholder groups – even if all expressed concerns about potential risks.

#### Who might not benefit?

Participants identified a broad range of factors that might at best limit the benefits a family could gain from an adapted intervention and at worst cause distress or conflict. These included: the behavioural variant of frontotemporal dementia (Ruth, LivDem facilitator and David, person living with dementia); evidence of abusive or aggressive behaviour (Sarah, LivDem facilitator and Alicia, psychotherapist); concurrent psychiatric diagnoses (Mia, LivDem facilitator); safeguarding issues (Leah, psychotherapist); and more generally the quality of the family relationship (Claudia, researcher), including families who cannot “*even sit in the same room*” with each other (Phoebe, LivDem facilitator). All of these pointed towards the importance for a thorough assessment before engaging with families and couples:

If you’re in a relationship which feels pretty unstable and there’s not a lot of trust around it’s maybe going to feel more threatening … So, I suppose the question might be to the couples ‘would you find it helpful to maybe talk a bit more about this experience together?’ (Leah, psychotherapist)

A number of participants were also clear that many people with dementia preferred not to think about what was happening, and that it would be important to ensure that the person living with dementia could bear to face difficult feelings and that they genuinely wanted to talk about their condition:

We had a volunteer from Age UK years ago who would come and spend time with mum, you know, take her to the shops, the whole time she was going ‘So why do I have her? Because I've got dementia?’. She wants to ignore it … it's the personal thing. (Eva, family supporter)

For many participants, any intervention would need to be offered at the right time – at a point when families were ready to begin to process the diagnosis and to gradually adjust to life with dementia:

What we see is that they need maybe a few weeks or months to start adjusting on their own to diagnosis before they can emotionally start talking about it with someone else. So, if the family, the dyad sessions are offered too soon, that might not work. (Mia, LivDem facilitator)

The requirement, then, to assess potential couples for their readiness to change places a high premium on the skills and abilities of the clinicians who will be delivering the adapted intervention.

### “It’s such a fine line”: The importance of empathetic and skilled facilitation

Given the range of issues that an adapted LivDem intervention would need to address, stakeholders also reflected on the personal and professional competencies that would be required to deliver this intervention and the wider context that would be necessary to support this work.

#### Skills and support

Participants identified a wide range of qualities for potential facilitators. This included: being “*curious about the work that they do in the context of dementia”* (Leah, psychotherapist); someone with “*confidence, experience”* and who is *“comfortable with the unknown”* (Phoebe, LivDem facilitator); who is “*very patient”* with *“a big bottle of kindness*” (Grace, person living with dementia); and with qualities including “*maybe kindness, curiosity, empathy, tentative gentle approach, not too on the nose, non-judgmental, honest”* (Nick, researcher).

The facilitator thus needs to carry the personal and professional qualities to engender trust and communicate compassion, whilst also conveying an honest understanding that empowers those they work with. James, the supporting partner for Grace, referred to their experiences of working with Liz, who had reframed Grace’s description of herself as *“being demented”* as instead *“living with dementia”*. In this way Liz was:

Being able to be positive … but still being real. So, to help a person to put it in my terms, although they’re living with dementia, don 't have the attitude I'm actually dying with dementia. Because you can look at it like that, you're either living with dementia or you're dying with dementia. (James, supportive partner)

Many participants, especially those who had facilitated LivDem, were aware of the need for facilitators to receive appropriate training:

Dealing with those upset relatives or service-users, you’d need a little bit of training on that. We are compassionate and you just want to put your arms around them and that’s not the right thing to do, so that training and just making sure you’re knowledgeable about what you’re delivering. (Tracey, LivDem facilitator).

Several participants described how the emotional strain of working with difficult feelings meant that good practice required a space for facilitators to find both supervision and a wider level of support:

Specifically, because it was quite complex work … things came up in that family setting that really you needed a lot of support around. (Janet, researcher)

The need for putative facilitators to embody skills that are often associated with counselling meant that there was a narrow boundary between maintaining the LivDem group course’s positioning as primarily a psychoeducational course, and the adapted version becoming more of a psychotherapeutic intervention. Thus, Leah (a psychotherapist) commented that there was: “*Such a fine line, acknowledging peoples’ need for defences … and [having] space to mourn, acknowledge the change or the loss”*.

#### Who is able to deliver?

In common with many group-based interventions, the current model of LivDem requires facilitators to work in pairs. Two participants, who were living with dementia, suggested having a co-facilitator who themselves was living with dementia:

I just wonder if there’s somebody who’s got dementia would find it helpful knowing that someone is there with them like that as well as the facilitator. (James, supportive partner)

In contrast, participants who were LivDem facilitators, researchers or psychotherapists generally saw facilitators as working within the health care system. Regardless of the person’s healthcare background, what was more important was that they showed certain personal and professional qualities such as willingness to learn and *“common sense”* (Alicia, psychotherapist). Some participants pointed toward roles in which people might not have had suitable training or skills to work comfortably with families in this way, for example dementia care advisors (Chloe and Josh, both LivDem facilitators) or psychology wellbeing practitioners (Charlie, psychotherapist). For David, a man living with dementia, the “*the label for the professional”* did not matter, rather “*what matters is the quality of the person and the quality of the training and the support they have”*.

### “You deal with it with your family”: how best to involve families

While providing a space for families to talk about the dementia more openly together might be beneficial, some stakeholders also had concerns. As described above (section 1.1), there was a risk that the carer would take the conversation “*over and* [the] *person with dementia potentially losing their voice*” (Janet, researcher). Similarly, Mia who as a LivDem facilitator was used to working in a group context where carers were excluded from the majority of sessions, emphasised the importance of ensuring that people with dementia were able to talk about their experiences away from their families:

They really needed the help of a professional to exchange their feelings, their emotions, their hopes, their desires, what they're frightened about for the future and they really needed a safe space where they could do so. (Mia, LivDem facilitator)

At the same time participants recognised that by excluding carers LivDem groups also risked shutting down discussion within families. We identified a number of different perspectives on how to balance these potentially conflicting frames of reference.

#### Alternative formats

Participants described different options for potential hybrid interventions which they hoped would combine the benefits of both individual sessions with family members and the safe space created by the group. Thus Phoebe (LivDem facilitator), suggested a format of a group composed of couples or family members which combined large group meetings for four or five couples, and then smaller parallel meetings of people with dementia and their partner. However, other participants (often psychotherapists) suggested that this might create challenging dynamics:

There’s an awful lot to contain in that, because you’re getting triggered by other couples, other peoples’ experiences, and you’re often very full up with your own. (Leah, psychotherapist)

Another option put forward was a group for carers and a separate group for people with dementia, as well as some time together. However, Charlie (psychotherapist) highlighted that this could bring up “all sorts of stuff up about othering and belonging... paranoia of what you’re left out of or being talked about”.

A third alternative that participants suggested focussed on moving away from a group format to work separately with individual families and couples. Individual meetings with families would enable bespoke tailoring for a particular family’s needs in order to: “help map out areas of conflict” (Ali, LivDem facilitator) and “work on communication difficulties” (Janet, researcher). A participant with dementia contrasted such a meeting with his own experiences of individual therapy in which:

I've had situations where things have been discussed with me which would have helped both of us, and I've not always felt confident and sure in retelling that to [partner]… but also sometimes what affects me would be helpful for [partner] to know and to be able to support me with. (David, person living with dementia)

Several LivDem facilitators suggested that working with couples or families could be integrated with the existing group based LivDem intervention:

We really do feel that ideally a family intervention could either follow the LivDem group or be a substitute to the LivDem group if families don't feel like participating. (Mia, LivDem facilitator)

#### Connected services

We also asked participants where the adapted version of LivDem might sit in a care pathway. For instance, whether this would be best within an NHS service, in the voluntary, community and social enterprises sector or within both. Participants told us that the intervention would need to be part of a pathway in which people living with dementia were situated within a wider community (Janet, researcher); signposted to other services (David, person living with dementia) such as meeting centres and memory cafes (Emma, researcher) and which was seen as confidential and trustworthy (Alicia, psychotherapist). The proposed course could then act as a paving stone, fitting into a network of other services:

LivDem-families would need to be embedded in a system with other support for staff delivering it to refer on, to get supervision, to be advised by other professionals … I think if it was, for example … discharge to an advisor and then back to GP, they could be quite isolated. (Lucy, researcher)

The primary concern of participants, then, was for the new intervention to be embedded within diagnostic and post-diagnostic services.

## Discussion

### Summary of results

Twenty-six participants were consulted about a potential adaptation of the existing LivDem group intervention to help couples and families talk together about how dementia affects them. Our stakeholders included: people living with dementia and their family supporters; current LivDem group facilitators; researchers; and couples psychotherapists working who work with people living with dementia. Participants agreed that an intervention which involved the person with dementia and their partner and/or other members of their family would be a useful addition to currently available post-diagnostic interventions. Given that some people living with dementia describe a sense of drifting away from the important people in their lives whilst struggling to share their feelings (van Wijngaarden et al., 2019), an adapted version of LivDem might enable the person’s voice to be heard by precisely those people who most need to hear it.

At the same time, any intervention needed to address a range of concerns – and importantly needed to be targeted only on those families who were ready to talk about the dementia and who would find this helpful. Those families where the intervention might be contra-indicated included people who had been diagnosed with a behavioural variant of frontotemporal dementia, who had concurrent or a significant pre-morbid history of mental health problems or where the quality of the familial relationships was low. It was also important to address issues around the capacity of the person with dementia to give informed consent to take part or more generally unwillingness to engage with the intervention.

### Supporting families to adjust to dementia

Many of the themes we identified in this study echo concerns that have been expressed previously in the literature. Thus, some previous approaches to couples work where one person has dementia have been criticised for lacking a genuine dyadic approach which values both partner’s views of the impact of the relationship equally (Bielsten and Hellstrӧm, 2019a) and in some cases have disregarded the voice of the person living with dementia entirely (Bielsten and Hellstrӧm, 2019b). In a similar vein, participants in this study emphasised the importance of supporting a sense of ‘togetherness’ within families within which they were able to share emotions, communicate more openly and discuss their differing needs, and in so doing potentially provide the anchor required to feel reconnected with family members again.

At the same time, the research literature suggests that there are a series of challenges in training the wider health and social workforce to deliver interventions targeting familial adjustment (Benbow & Sharman, 2014). One solution suggested in this study was that the adapted version of LivDem should make use of a blended ‘toolbox’ approach, including therapeutic, psychoeducational and practical elements. This should seek to equip facilitators with qualities including conveying understanding, warmth and a certain curiosity into the lived experience of families including their similarities and differences. Good supervision, training and support were seen as essential in ensuring effective delivery of the intervention. Of the different formats suggested by participants, there was a preference towards working with individual families apart from the group and having the flexibility to tailor the content to meet the specific needs of different families. As such this intervention might sit in a service as an addition to the current group format, either before this occurred to ease people into the course, or as a follow-up once the group had finished or indeed as an entirely independent alternative to group interventions. This might sit within different service settings including both statutory and voluntary sectors.

Despite these challenges, finding a way to engage couples and families in adjusting to dementia is vital precisely because health and social practitioners rarely have a choice of *whether* or not to work with the wider relationships in a person’s life – but instead have to choose *how* to do so (Cheston, 2022). Given both the importance of psychological support after a diagnosis to people living with dementia (Bamford et al., 2021) and its relative lack (Royal College of Psychiatrists, 2022), it is therefore important to identify ways in which such support can be widely provided.

### Limitations and strengths

A potential strength of a future LivDem-Families intervention would be that it is grounded in an existing intervention that supports participants to adjust to dementia and based around the principles of space (for people living with dementia to talk about their condition) and occurs at a pace that works for them. However, there are a number of important limitations. While we consulted with a wide range of stakeholders, most (21 of 26 participants) were professionals (clinicians with experience of delivering LivDem, psychotherapists and academics) and thus whilst the voices of people living with dementia and their families were included, these were not the sole focus of the research. Moreover, our participant sample was limited, and we did not identify voices from people belonging to minority groups, including those from marginalised ethnic communities or the LGBTQ + community. This may mean these results are not representative of the views of these communities and thus the results reported here may not be transferable across a range of caregiving dyads.

Finally, this research was predicated on the assumption that talking about one’s experience of living with dementia is helpful for many people, and it is likely that stakeholders who took part in this research agreed with this premise. However, we acknowledge that for some this is contentious issue, and that some research suggests that greater awareness can be associated with worse outcomes (e.g., Alexander et al., 2021).

### Future research

Stakeholders in this research overwhelmingly agreed that adjusting to a dementia diagnosis is challenging for people living with dementia and their family members. Therefore, future research needs to pay greater attention to how people adjust to dementia and services need to recognise this challenge and support families in this process. The LivDem model, delivered either to a group or as proposed here to individual families, offers a practical intervention which can be delivered in the UK health or voluntary sectors by non-psychologists. In light of the results of this research, we will now focus on adapting the LivDem model for couples and families and exploring the feasibility of this intervention in supporting families affected by dementia.

## Notes

1. Approval was received on 19th August 2022 (HAS.22.07.140).

2. Reasonable requests for access to data in the form of transcripts of interviews and workshops can be made directly to the authors.

## Appendix 1 Focus Group Topic Guide

For each group:

• Introduction to research and the LivDem model
• Explain function of the focus group, why are we convening it and what is their task
• For groups b) and c) researcher to explain a bit about LivDem groups including broad themes/topics covered

### Group a) existing LivDem facilitators

1. Ice-breaker – introducing themselves, where they run their groups, how many groups they have run and for how long
2. What do you feel are some of the important elements within the LivDem course that helps people to open up about living with dementia?
3. Are there times in the group sessions when you feel family members would benefit from being there? If so, why? Which current LivDem sessions/elements might work well within this context?
4. Are there LivDem sessions that would not translate well to families? Are there sessions that would need adapting? *(A: for the ‘Intervention’ domain of CFIR)*
5. What would you feel comfortable discussing with families and what would feel out of your remit?
6. Are session handouts helpful to people living with dementia in LivDem in promoting conversations with their families? *(A: for the ‘Intervention’ domain of CFIR)*
7. What would the advantages and disadvantages be of implementing LivDem-Families in services? *(A: for the ‘Intervention’ domain of CFIR)*
8. What resources are needed for services to implement LivDem-Families successfully? *(A: for the ‘Intervention’ domain of CFIR)*
9. What factors or characteristics of people living with dementia and their families might affect their ability to engage in LivDem-Families? (*B: for the ‘Outer Setting’ CFIR domain; D for the ‘Individual’ CFIR domain)*
10. Are there any reasons why some people living with dementia would not benefit from LivDem-Families, or might even be at risk of harm? *(B: for the ‘Outer Setting’ CFIR domain; C: for the ‘Inner Setting’ CFIR domain)*
11. Are people living with dementia and their families likely to be able to stay engaged with LivDem-Families? How might a facilitator manage this? *(D for the ‘Individual’ CFIR domain)*
12. What extra training and support such as supervision would facilitators require to be able to run LivDem-Families? (*D for the ‘Individual’ CFIR domain)*
13. What would be the best way to deliver training for LivDem-Families facilitators? (*D for the ‘Individual’ CFIR domain) – would online training be appropriate?*
14. How can distressed family members be supported in LivDem-Families? (*E for the ‘process’ domain)*
15. Do services have the means to support LivDem-Families implementation? (*E for the ‘process’ domain)*
16. Are there any final thoughts you’d like to mention before we close?

### Group b) Researchers and Therapists

1. Ice-breaker – introducing themselves/their roles, how they got into the field of dementia, what areas of research do they have which might be especially relevant
2. What do you think might be important for family members to discuss together when one partner is diagnosed with dementia? What might stop them from having these discussions?
3. What would the advantages and disadvantages be of implementing LivDem-Families in services? (*A: for the ‘Intervention’ domain of CFIR)*
4. In your experience, would people living with dementia and their families participate in LivDem-Families if it was available to them? What in your opinion, might influence this decision? *(B: for the ‘Outer Setting’ CFIR domain)*
5. Are there other similar interventions are available to people living with dementia after diagnosis? *(B: for the ‘Outer Setting’ CFIR domain)*
6. What factors or characteristics of people living with dementia and their families might affect their ability to engage in LivDem-Families? What barriers might prevent people living with dementia and their families from accessing LivDem-Families? *(B: for the ‘Outer Setting’ CFIR domain)*
7. Are there any reasons why some people living with dementia would not benefit from LivDem-Families, or might even be at risk of harm? *(B: for the ‘Outer Setting’ CFIR domain; C: for the ‘Inner Setting’ CFIR domain)*
8. What factors would affect the likelihood that people living with dementia and their families would attend LivDem-Families? (*C: for the ‘Inner Setting’ CFIR domain)*
9. Would session handouts be helpful to people living with dementia in LivDem-Families in promoting conversations with their families? (*A: for the ‘Intervention’ domain of CFIR)*
10. What organisational policies or incentives could support implementation of LivDem-Families? *(B: for the ‘Outer Setting’ CFIR domain)*
11. Are people living with dementia and their families likely to be able to stay engaged with LivDem-Families? How might a facilitator manage this? *(D for the ‘Individual’ CFIR domain)*
12. How can distressed family members be supported in LivDem-Families? (*E for the ‘process’ domain)*
13. What extra training and support, such as supervision, would facilitators require to be able to run LivDem-Families? *(D for the ‘Individual’ CFIR domain)*
14. Are there any issues that may be too complex for LivDem facilitators to feel competent facilitating? Equally, are there any difficult conversations that facilitators can be encouraged to have, but which they may feel reluctant to do so?
15. Are there any final thoughts you’d like to mention before we close?

### Group c) People Living with dementia and Their Families

1. Ice-breaker – introducing themselves and/or their family members.
2. Do you feel it is important to talk about dementia as a family? What do you feel is important to discuss openly together? Are there issues that you would not feel comfortable discussing?
3. Are there areas you would feel you would benefit from having a third party (facilitator) to help you discuss with each other?
4. What areas do you feel would belong more in a counselling/therapy context?
5. What aspects of LivDem would need to be different in order for it to be successfully implemented with families and couples? (*A: for the ‘Intervention’ domain of CFIR)*
6. Which current LivDem sessions might work well and which would need adapting? (*A: for the ‘Intervention’ domain of CFIR)*
7. Would you find session handouts helpful in promoting conversations with your family? (*A: for the ‘Intervention’ domain of CFIR)*
8. Would you participate in LivDem-Families if it was available to you? What would influence this decision? *(B: for the ‘Outer Setting’ CFIR domain)*
9. Do you know of any other dementia interventions that you would feel would be preferable to families living with dementia after diagnosis? *(B: for the ‘Outer Setting’ CFIR domain)*
10. What factors or characteristics might affect someone’s ability to engage in LivDem-Families? *(B: for the ‘Outer Setting’ CFIR domain)*
11. Are there any reasons why some people living with dementia would not benefit from LivDem-Families, or might even be at risk of harm? *(B: for the ‘Outer Setting’ CFIR domain)*
12. What barriers might prevent people living with dementia and their families from accessing LivDem-Families? *(B: for the ‘Outer Setting’ CFIR domain)*
13. What factors would affect the likelihood that people living with dementia and their families would attend LivDem-Families? (*C: for the ‘Inner Setting’ CFIR domain)*
14. What would be the most important benefits of LivDem-Families? (*C: for the ‘Inner Setting’ CFIR domain)*
15. How can distressed family members be supported in LivDem-Families? *(E: for the ‘process’ domain)*
16. How can services support people living with dementia and their families to engage in LivDem-Families? *(E: for the ‘process’ domain)*
17. Are there any final thoughts you’d like to mention before we close?
